# Acute effects of physical exercise on the serum insulin-like growth factor system in women with fibromyalgia

**DOI:** 10.1186/s12891-017-1402-y

**Published:** 2017-01-25

**Authors:** Kaisa Mannerkorpi, Kerstin Landin-Wilhelmsen, Anette Larsson, Åsa Cider, Olivia Arodell, Jan L. Bjersing

**Affiliations:** 1 Institute of Neuroscience and Physiology, Section of Health and Rehabilitation, Sahlgrenska Academy, University of Gothenburg, Gothenburg, Sweden; 2 Sahlgrenska University Hospital, Physiotherapy and Occupational therapy, Göteborg, Sweden; 3 Department of Rheumatology and Inflammation Research, Institute of Medicine, Sahlgrenska Academy, University of Gothenburg, Guldhedsgatan 10, Box 480, 40530 Göteborg, Sweden; 4 Section of Endocrinology, Sahlgrenska Academy, Sahlgrenska University Hospital, Institution of Medicine, University of Gothenburg, Gothenburg, Sweden; 5 Sahlgrenska University Hospital, Rheumatology, Göteborg, Sweden

**Keywords:** Exercise, IGF, Fibromyalgia, Fatigue, Pain

## Abstract

**Background:**

Increased Serum insulin-like growth factor-1 (S-IGF-1) has been noted after physical activity in healthy subjects, while the acute release of S-IGF-1 in relation to exercise has not previously been studied in women with fibromyalgia (FM). S-IGF-1 and its binding protein (S-IGFBP-3) are mediated by growth hormone and have anabolic effects on the skeletal muscle. Aim of the study was to investigate acute release of IGF-1 after aerobic exercise in women with FM.

**Methods:**

The acute effect of physical exercise on S-IGF-1 and S-IGFBP-3 were studied in 22 women with FM and in 27 healthy controls during moderate and high-intensity cycling (i.e. ratings 12–13 and 15–17, on Borg’s perceived exertion scale (RPE), respectively). Self-reported pain and fatigue were recorded. Differences within and between the two groups were analyzed.

**Results:**

After 15 min of bicycling, S-IGF-1 and S-IGFBP-3 increased both within the group with FM and in the healthy controls (*p* < 0.01). The increases in S-IGF-1 did not significantly differ between the women with FM and the healthy control group (mean increase 11 ± 10 vs. 11 ± 15 ng/ml and 13 ± 10 vs. 19 ± 22 ng/ml) when bicycling at moderate or high intensity, respectively. Self-reported pain and fatigue during exercise, irrespective of intensity, were higher in women with FM compared with healthy controls (*p* < 0.001).

**Conclusions:**

Fifteen minutes bicycling at moderate intensity was sufficient to acutely mobilise S-IGF-1 in women with FM similarly to healthy controls in spite of higher score of fatigue and pain in women with FM. Hence, patients with FM were able to activate their skeletal muscle metabolism during a short, moderate bout of exercise and were not resistant to training effects. The result is important for encouraging clinical rehabilitation of patients with FM who commonly exercise at a moderate, rather than at a high-intensity level.

**Trial registration:**

ClinicalTrials.govNCT01592916, May 4, 2012.

## Background

Fibromyalgia (FM) is characterised by persistent widespread pain and increased pain sensitivity and tenderness [1], and is associated with impaired physical fitness [2]. Pain in FM is attributed to central sensitization and impaired pain inhibition [3], implying that the pain increases during physical activities. Activity-induced pain in FM [4] is a probable reason why persons with FM commonly prefer physical activities of low, rather than of high intensity [5, 6].

Studies have shown that physical capacity can be improved through exercise in patients with FM [7]. There are, however, several factors, such as age, sex, health and hormonal status, inflammation and nutrition that can influence the effect of exercise. Growth hormone has been of special interest in research of FM, due to documented disturbance in the pituitary growth hormone axis in FM, especially in persons with severe symptoms [8, 9]. Growth hormone is influenced by several factors, such as sleep, stress, nutrition, smoking, body composition and physical activity [10]. Growth hormone exerts its effect via the insulin-like growth factor (IGF) system in the liver including total and free IGF-,1 IGF-2 and six binding proteins (IGFBP) of which IGF-1 and IGFBP-3 are most important in the skeletal muscle [11] with positive outcomes of exercise [12] . Furthermore, exercise has beneficial effects in the central nervous system via uptake of peripheral IGF-1 [13]. S-IGF-1 increased in lean women with FM, but not in overweight counterparts, after aerobic exercise twice weekly during 15 weeks [14].

Acute release of IGF-1 in relation to exercise has not previously been studied in women with FM. The aim was to study the effects of short bouts of aerobic exercise of moderate and high intensities, respectively, on S-IGF-1 in women with FM in comparison with healthy controls.

## Methods

### *Study design*: an experimental study

#### Study population

Women with FM, aged 20 to 50 years, and fulfilling the American College of Rheumatology criteria for FM [1] and healthy women without FM, aged 20 to 50 years, were eligible for the exercise intervention. Eligible patients were identified among those having participated in previous studies, contacted by telephone and asked about their interest of participating in the study. Healthy controls were recruited among employees at the hospital and university settings to participate in the same exercise tests. All patients and controls should be able to participate in two bicycle tests at 8–12 o’clock at the 5–7th day of the menstrual cycle.

Exclusion criteria were concomitant severe diseases hindering participation in the exercise tests, such as high blood pressure, a cardiovascular disease, severe osteoarthritis, psychiatric disease.

#### Study procedures

The study protocol was described, days for planned examinations were presented, and if matching the volunteer’s schedule, the first test occasion was booked. A patient information sheet was sent to the volunteers. Participation involved two visits and the participants were told to reserve 1.5 h for each visit. The two exercise tests were separated by a month. The tests were conducted during mornings at the 5–7th day of the menstrual cycle, to control for the hormonal status. Subjects not being able to return after 1 month were offered possibility to do the test after 2 months. Subjects completing both tests were included in the statistical analyses.

Before the examination, verbal information was given and an informed consent was signed by the test person and the nurse. The first blood test was sampled in the fasting state, after which a light standardized breakfast (a cheese sandwich, caffeine-free coffee or tea) was served. Information about demographic data and pharmacological treatment was gathered by an interview, and the test person was asked to fill in a questionnaire about health status, symptoms and physical activity habits.

*Body composition* was evaluated with body weight (kg) and height (m) in light clothes without shoes for calculation of body mass index (BMI) as body weight divided by height squared (kg/m^2^). Furthermore, bioimpedance, (SEAC Multiple frequency bioimpedance meter model SFB 2, UniQuest Ltd, Queensland, Australia) was performed. This method is based on resistance and reactance in the total body. The method measures the intracellular and extracellular resistance and the body fat and fat-free or lean mass can be derived in relation to height, body weight and age [15, 16].

*The number of tender points* to verify the ACR criteria [1] for FM was calculated.

## The two exercise tests on an ergometer cycle

The exercise tests were conducted on a Monark ergometer cycle model 829 E (Monark, Vansbro, Sweden) and the load for each patient was chosen after an interview regarding the participant’s physical activity level to fit a given level on the Borg’s scale of perceived exertion (RPE scale) [17]. Heart rate, blood pressure and rate of perceived exertion [17] were monitored during the test. After 15-min of ergometer cycle test, the second blood test was sampled, after which the test person was asked to rate her pain and fatigue. Similar examinations were conducted after 30 min of rest (0–45 min).

### Ergometer cycle test of moderate and high intensities

A 15-min test on a ergometer bicycle [18] was performed and the participant was asked to rate her exertion on RPE scale. Moderate intensity was defined as 12–13 and high 15–17 on the RPE-scale, respectively [17]. During the first test the patients also mentally adjusted themselves to manage the second test.

### Exercise load

The load (watt) during ergometer cycling was monitored every other minute and adjusted to fit the participant’s perceived exertion level: i:e: 12–13 or 15–17, respectively, on the RPE-scale. The loads (watts) during the test performance were transformed to kilojoules to represent the total work during the test performance.

*Pain threshold* was examined by using an algometer (Somedic Production AB, Sollentuna, Sweden), measured in kilopascals (kPa) [19]. The pain threshold was measured in two tender-point locations in the upper and lower extremities, respectively: musculus trapezius, musculus supraspinatus, musculus gluteus and medial fat pad of the knee [1] . The mean value was applied, and a higher value indicated a better health.

*Pain and fatigue* were rated on a visual analogue scale (0–100; low to high). A higher score indicates more severe pain or fatigue.

*Health status* was assessed by the Fibromyalgia Impact Questionnaire (FIQ), a higher score indicating more severe impairment [20].

### Blood sampling

Serum samples were collected from the cubital vein in the fasting state at rest at baseline (0), after 15 min cycling and 30 min of rest after the bicycling was terminated (45 min). Biological markers were analyzed by sandwich enzyme-linked immunosorbent assays (ELISAs) using a pair of specific antibodies for human interleukin 8 (M1918, 1.0 pg/ml, Sanquin reagents, Amsterdam, the Netherlands). ELISAs were read with a Spectramax 340 from Molecular Devices (Sunnyvale, CA, USA). Human high sensitivity C-reactive protein (sCRP, ref < 5 μg/ml) was analyzed by immunoturbidimetic analysis by the accredited Laboratory for Clinical Chemistry at the Sahlgrenska University Hospital, Gothenburg, Sweden. Total S-IGF-1 was measured by solid-phase, enzyme-labeled chemoluminescent immunoassay with IDS-iSYS IGF1 immunoassay (IS-3900, Immunodiagnostic Systems Boldon, UK), S-IGFBP-3 and free IGF-1 with reagents from DY675, RnD Systems, Minneapolis, MN, USA.

### Statistics

Non-parametric statistic tests were chosen due to small sample sizes. Descriptive data were presented as mean and SD or median and range. Differences within a group were analyzed by the Wilcoxon signed rank test and between the groups with Mann Whitney *U*-test. Fischer’s exact test was applied in the analysis of categorical data. Relations between the variables were examined with the Spearman’s correlation test. All significant tests were two-tailed, and values of *p* < 0.05 were considered significant.

## Results

### Study population

A total of 28 women with FM were screened for the study. Three of them did not fulfill the American College of Rheumatology 1990 criteria for FM and one had a high blood pressure, leaving 24 patients. Two patients withdrew themselves from the study due to time limitations. Thus, the patient population comprised 22 women with FM, with the mean age of 44 (range 33–50) years. The healthy reference group for the exercise tests comprised 27 women, mean age 44 years (range 29–50) years (ns). Anthropometric and background data are given in Table 1.

**Table 1:** Background data for women with fibromyalgia (FM) and the healthy reference group

	Women with FM, *n* = 22			Reference group, *n* = 26			*p*-value
	Mean	SD	Range	Mean	SD	Range	
Age, year	44	4	33–50	44	5	29–50	0.808
BMI, kg/m^2^	28.2	5.3	20.5–38.9	23.2	2.7	18.8–31.2	<0.001
Fat, kg	28.3	12.0	11–51	17.6	8.0	4–40	0.001
Fat free mass, kg	47.8	7.0	38–63	47.9	5.4	39–60	0.695
Tender points, 0–18	13	1.8	11–16	0	0	0	<0.001
Pain localisations, 0–18	13	3.4	6–18	1.0	1.4	0–5	<0.001
Algometry, kPa	255	56	167–403	378	107	173–566	<0.001
Smoking, yes^a^	*N* = 2		0–2	*N* = 0		0	0.186
Sleep quantity, 1–4	2.8	0.9	1–4	2.1	0.6	1–3	0.003
Sleep quality, 1–4	2.6	0.7	1–4	1.6	0.6	1–3	<0.001
Work hours, 0–40	27	10.5	0–40	37	5.0	20–40	<0.001
FIQ total score, 0–100	48	16	10–73	9	9	0–28	<0.001
Hand force, maximum, right hand, N	215	83	67–381	252	60	130–396	0.129
LTPAI, hours/week	5.3	3.5	1–17	7.0	4.2	1–16	0.170
LTPAI, hard, hours/week	0.6	1.3	0–6	1.4	1.4	0–6	0.024

*FIQ* Fibromyalgia Impact Questionnaire, *LTPAI* Leisure Time Physical Activity Instrument, Algometry = pain threshold

^a^Information from one subject with FM was missing

### Exercise performance at moderate intensity

#### Rating of perceived exertion (RPE)

No significant difference was found for perceived exertion between the two groups. The mean scoring at 15-min of cycling was 14.9 ± 1.6 in the FM-group, while it was 14.2 ± 1.9 in the reference group (*p* = 0.310).

*Exercise load* in women with FM at 15 min was 48 ± 20 Watt (W), while it was 94 ± 27 W in the reference group (*p* < 0.001). The heart rate at 15 min was 114 ± 16 beats/min in those with FM and 137 ± 18 beats/min in the healthy group (*p* < 0.001). Total work in the group with FM at 15 min was 34 ± 11 kJ (kJ), while it was 66 ± 12 kJ in the reference group (*p* < 0.001).

### Exercise performance at high-intensity

#### Rating of perceived exertion (RPE)

No significant difference was found for perceived exertion between the two groups. The mean RPE was 17.3 ± 1.6 in the FM-group and 16.6 ± 1.5 in the healthy group (*p* = 0.85).

*Exercise load* in the group with FM at 15 min was 93 ± 25 W, while it was 127 ± 28 W in the healthy control group, implying that the latter group exercised at a significantly higher work load (*p* < 0.001). The heart rate at 15 min was 147 ± 20 beats/min in the FM-group and 160 ± 18 beats/min in the healthy group (*p* = 0.028). Total work in the FM-group at 15 min was 62 ± 15 kJ, while it was 91 ± 17 kJ in the reference group (*p* < 0.001). Total work was significantly higher in high intensity compared to moderate intensity both in the FM–group (*p* < 0.001) and in the reference group (*p* < 0.001).

### Change in S-IGF-1

S-IGF-1 was similar in women with FM and controls at baseline and increased similarly in both groups, during the two exercise intensities. Mean response of S-IGF-1 was 6% in patients with FM during both the moderate and the high-intensity exercise. Mean response of IGF-1-t in the reference group was 7% during the moderate intensity exercise and 10% during the high-intensity exercise (ns between patients and controls), (Tables 2 and 3).

**Table 2:** Values obtained at moderate intensive exercise performance in women with fibromyalgia (FM) and the healthy reference group. Mean value and SD for baseline values, differences (∆), *p*-value for within-group differences as well as for between- group differences are presented

Moderate intensity exercise performance								
	Women with FM, *n* = 22			Healthy reference group, *n* = 27			Between-group differences	
	0 min	15 min–0 min		0 min	15 min–0 min		0 min	Change 0–15 min
	Mean ± sd	∆ mean ± sd	*p*-value	Mean ± sd	∆ mean ± sd	*p*-value	*p*-value	*p*-value
S-IGF-1 (ng/ml)	178 ± 58	11 ± 10	<0.001	179 ± 69	13 ± 10	<0.001	0.825	0.629
free IGF-I (ng/ml)	3.5 ± 1.6	−0.15 ± 0.34	0.014	4.0 ± 2.09	−0.38 ± 0.47	<0.001	0.457	0.119
S-IGFBP3 (ng/ml)	428 ± 44	11 ± 16	0.007	426 ± 37	14 ± 19	0.001	0.880	0.662
sCRP (μg/ml)	2.0 ± 2.2	0.14 ± 0.18	0.001	0.56 ± 0.48	0.35 ± 0.46	<0.001	<0.001	0.215
IL-8 (pg/ml)	9.2 ± 4.5	−5.9 ± 1.6	0.105	9.6 ± 2.5	−0.3 ± 1.0	0.077	0.769	0.600
Pain (0–100 mm)	40 ± 19	7.6 ± 16	0.044	0.8 ± 2.1	2.0 ± 6.8	0.093	<0.001	0.015
Fatigue in legs (0–100 mm)	26 ± 23	28 ± 24	<0.001	1 ± 2.4	6.0 ± 8.5	0.002	<0.000	<0.001
Fatigue global (0–100 mm)	51 ± 27	9.2 ± 17	0.038	4 ± 6.4	10 ± 12	0.002	<0.001	0.672

*S-IGF-1* serum insulin-like growth factor-1, *S-IGFBP3* Insulin-like growth factor binding protein-3, *sCRP* human high sensitivity C-reactive protein, *IL-8* interleukin-8

**Table 3:** Values obtained at high-intensity exercise performance in women with fibromyalgia (FM) (*n* = 22) and the healthy reference group (*n* = 27). Mean value and SD of differences (∆), *p*-value for within-group differences as well as for between- group differences are presented

High-intensity exercise								
	Women with FM, *n* = 22			Healthy reference group, *n* = 27			Between-group differences	
	0 min	15 min–0 min		0 min	15 min–0 min		0 min	Change 0–15 min
	Mean ± sd	∆ mean ± sd	*p*-value	Mean ± sd	∆ mean ± sd	*p*-value	*p*-value	*p*-value
S-IGF-1 (ng/ml)	195 ± 60	11 ± 15	<0.001	181 ± 66	19 ± 22	<0.001	0.244	0.560
Free IGF-1 (ng/ml)	4.7 ± 2.6	−0.42 ± 0.48	0.014	4.7 ± 3.2	−0.56 ± 0.76	<0.001	0.755	0.843
S-IGFBP3 (ng/ml)	556 ± 75	25 ± 37	0.009	614 ± 91	25 ± 41	0.008	0.037	0.942
sCRP μg/ml)	2.1 ± 2.4	0.06 ± 0.16	0.002	0.70 ± 0.77	0.04 ± 0.06	<0.001	0.001	0.119
IL-8 (pg/ml)	7.5 ± 3.0	0.7 ± 2.9	0.545	9.7 ± 4.2	−0.2 ± 1.9	0.673	0.107	0.565
Pain (0–100, mm)	46 ± 24.4	−4.0 ± 16.1	0.291	2.4 ± 7.4	0 ± 3.6	0.878	<0.001	0.139
Fatigue in legs (0–100 mm)	31 ± 21	28 ± 20	<0.001	1.2 ± 3.1	12 ± 13	<0.001	<0.001	0.003
Fatigue global (0–100 mm)	50 ± 24	8.4 ± 19	0.007	3.0 ± 5.3	6.3 ± 10	<0.001	<0.001	0.414

*S-IGF-1* serum insulin-like growth factor-1, *S-IGFBP3* Insulin-like growth factor binding protein-3, *sCRP* human high sensitivity C-reactive protein, *IL-8* interleukin-8

### Change in S-IGFBP-3and free IGF-1

S-IGFBP-3 and free IGF-1 were similar in the two groups of women at baseline. S-IGFBP-3 increased and free IGF-1 decreased significantly during the two exercise intensities in women with FM and in controls (ns between groups), (Tables 2 and 3).

### Change in IL-8 and sCRP

Serum IL-8 was similar in women with FM and controls at baseline and did not change during the exercise in any of the two groups (Tables 2 and 3).

sCRP levels were higher in women with FM compared with controls at baseline. sCRP increased during both exercise intensities in both groups (ns between groups), (Tables 2 and 3).

### Body composition

Body mass index and body fat were higher in women with FM. However, lean mass was similar (Table 1). Lean mass did not correlate with S-IGF-1 (*p* = 0.200) in any group.

In women with FM, body fat was correlated with pain (*r* = 0.434, *p* = 0.044, *n* = 22) and global fatigue (*r* = 0.57, *p* = 0.006, *n* = 22) at 15 min of high intensity exercise and the level of sCRP (*r* = 0.487, *p* = 0.022, *n* = 22). BMI correlated with global fatigue at 15 min of exercise (*r* = 0.514, *p* = 0.014, *n* = 22) and sCRP level (*r* = 0.465, *p* = 0.029, *n* = 22) and tended to correlate negatively with baseline levels of S-IGF (*r* = −0.415, *p* = 0.055) in women with FM. BMI or body fat did not correlate with change in S-IGF-1 at moderate or high intensity exercise, respectively, in women with FM.

### Pain

Patients with FM reported more pain at baseline when compared to the reference group (*p* < 0.001). Pain increased more in the women with FM during the moderate intensity exercise (Table 2), while there was no difference for change between the two groups during the high-intensity exercise (Table 3).

### Pain threshold

Women with FM had significantly lower pain threshold measured with algometry at baseline, on both exercise occasions, when compared to the reference group (Table 1). No significant difference was found (*p* = 0.66) when the baseline pain thresholds at the first and the second test occasion were compared within the FM-group. Pain thresholds decreased in the FM-group after moderate (−25 ± 20, *p* < 0.0001) and tended to decrease after high intensity exercise (−12 ± 28, *p* = 0.08). Pain thresholds increased after high (17 ± 38, *p* = 0.02) but not after moderate intensity exercise in controls.

### Fatigue in the legs, global fatigue

Women with FM reported more fatigue in the legs as well as global fatigue at baseline when compared to the reference group (*p* < 0.001). Fatigue in the legs increased during both exercise intensities in both groups and was more pronounced in the FM-group than in the reference groups. Global fatigue increased in both groups during both exercise intensities, (ns between the two groups), (Tables 2 and 3).

## Discussion

Growth hormone derived S-IGF-1 and its binding protein S-IGFBP-3 increased after moderate exercise during 15 min on an ergometer cycle in women with FM similar to women in the healthy reference group in spite of more reported pain and fatigue in FM. The mean increase in S-IGF-1 was in line with previous reports from healthy individuals [21, 22].

Exercise mobilises systemic IGF-1 from the liver and locally in skeletal muscle. Growth hormone is the main stimulus for hepatic IGF-1 production in the resting state but there is a delay of several hours of growth hormone-mediated release of hepatic IGF-1 post-exercise [23, 24]. This indicates that the increase in S-IGF-1 in this study was growth hormone-independent. It has been shown that circulating IGF-1 is mobilised from active muscles during intense exercise [11, 25].

A higher level of S-IGF-1 has been associated with better physical fitness among both younger [26] and older individuals [27]. In healthy women, IGF-1 and ratio of IGF-1/IGFBP-3 were associated with maximum power output [28]. Resting level of S-IGF-1 declines with aging together with sarcopenia and osteopenia [10, 29], and it is low in chronic and severe diseases [30]. An increase of S-IGF-1, ranging from ~10 to 30%, has been measured after 5–10 min of high-intensity bouts of exercise in healthy individuals [21]. Mechanisms regulating the concentration of IGF-1 are complex, and the transient increase during a bout of exercise returns to baseline within one hour [21]. This may involve uptake into peripheral tissues [31] and uptake into the central nervous system [32].

The reduced levels of free IGF-1 may be due to activity induced uptake of IGF-1 from the blood. IGF-1 is actively taken up from blood into the central nervous system via the blood brain barrier and via the choroid plexus. Uptake of IGF-1 over the blood brain barrier is stimuli-dependent and coupled with increased neuronal activity [32]. Both aerobic exercise and systemic injection of IGF-1 increase cerebrospinal levels of IGF-1 without alterations in serum levels in rats [13]. It has therefore been suggested that serum levels of IGF-1 may be rapidly normalized due to strong uptake into the brain [33] and IGF-1 uptake may be increased by the physical activity. There is also evidence of re-uptake of IGF-1 into peripheral muscle after exercise [31]. The present study would indicate that both mobilisation of IGF-1 into the blood and possible active re-uptake into tissues after exercise is preserved in FM patients. As expected, the patients with FM showed a lower pain threshold [1] and higher ratings of pain and fatigue in legs and global fatigue at baseline. A notable increase of local fatigue was found already during exercise at moderate intensity. This finding is in line with a previous study reporting that physical exercise induces fatigue in FM [34]. The lean mass, which mainly reflects the skeletal muscle mass, was similar in both groups of the present study. However, BMI and body fat content were higher in the patients than the controls. This might contribute to the higher self-reported fatigue, during exercise. In a previous study, increased levels of S-IGF-1 after 15 weeks of aerobic exercise was found in lean but not in overweight and obese patients with FM [14]. Furthermore, BMI was associated with CRP in FM. This implies a peripheral low-grade inflammation and could potentially contribute to reduced muscular IGF-1 response in overweight women with FM [35–37]. However, in the present study, BMI did not correlate with the acute IGF-1 response.

The exercise load was defined by means of the RPE scale, a method recommended for determining exercise intensity [38]. The ratings of exertion showed that both groups followed the protocol at the two test occasions, without any significant differences for the rating of exertion, and therefore the groups were comparable. Walking on flat ground indoors with a velocity of 4 km/h for a person with a bodyweight of 70 kg corresponds to approximately 50 W. All the participants were able to carry on a normal conversation without any notable impact on their breathing rate. Thus, the exercise load corresponded to a moderate exercise level. In women with FM, the exercise load remained at 50 W also at the end of the exercise period.

Previous studies have shown that women with FM have a lower muscular physical fitness when compared to healthy women [2, 39]. This might explain why women with FM cycled at a significantly lower exercise load than the healthy reference group in the present study. The patients with FM also reported less hours spent on vigorous exercise during their leisure time, compared to the reference group, in line with previous studies [6].

## Conclusions

Fifteen minutes of exercise at moderate intensity was sufficient to achieve an increase in total S-IGF-1 and its binding protein, S-IGFBP-3. Hence, patients with FM were able to activate their skeletal muscle metabolism during a short, moderate bout of exercise, despite of their pain and fatigue. This knowledge is of importance for clinical rehabilitation of patients with FM who commonly exercise at this level, rather than at a high intensity level.

## Abbreviations

BMI: Body mass index
FIQ: Fibromyalgia impact questionnaire
FM: Fibromyalgia
free IGF-1: Free insulin-like growth factor-1
IGFBP-3: Serum insulin-like growth factor binding protein-3
IL-8: Interleukin-8
kJ: Kilojoules
kPa: Kilopascal
RPE: Reported perceived exertion
sCRP: Serum C-reactive protein
S-IGF-1: Serum total insulin-like growth factor-1
W: Watt
